# Nonalcoholic fatty liver disease is associated with dysbiosis independent of body mass index and insulin resistance

**DOI:** 10.1038/s41598-018-19753-9

**Published:** 2018-01-23

**Authors:** Hannah E. Da Silva, Anastasia Teterina, Elena M. Comelli, Amel Taibi, Bianca M. Arendt, Sandra E. Fischer, Wendy Lou, Johane P. Allard

**Affiliations:** 1 Sunnybrook Health Sciences Centre, Toronto, Ontario Canada; 2 Department of Medicine, Toronto General Hospital, University Health Network, University of Toronto, Toronto, Ontario Canada; 3 Department of Nutritional Sciences, University of Toronto, Toronto, Ontario Canada; 4 Dalla Lana School of Public Health, University of Toronto, Toronto, Ontario Canada

## Abstract

This study aimed to determine if there is an association between dysbiosis and nonalcoholic fatty liver disease (NAFLD) independent of obesity and insulin resistance (IR). This is a prospective cross-sectional study assessing the intestinal microbiome (IM) of 39 adults with biopsy-proven NAFLD (15 simple steatosis [SS]; 24 nonalcoholic steatohepatitis [NASH]) and 28 healthy controls (HC). IM composition (llumina MiSeq Platform) in NAFLD patients compared to HC were identified by two statistical methods (Metastats, Wilcoxon). Selected taxa was validated using quantitative PCR (qPCR). Metabolites in feces and serum were also analyzed. In NAFLD, 8 operational taxonomic units, 6 genera, 6 families and 2 phyla (Bacteroidetes, Firmicutes) were less abundant and; 1 genus (*Lactobacillus*) and 1 family (Lactobacillaceae) were more abundant compared to HC. Lower abundance in both NASH and SS patients compared to HC were confirmed by qPCR for *Ruminococcus, Faecalibacterium prausnitzii* and *Coprococcus*. No difference was found between NASH and SS. This lower abundance in NAFLD (NASH+SS) was independent of BMI and IR. NAFLD patients had higher concentrations of fecal propionate and isobutyric acid and serum 2-hydroxybutyrate and L-lactic acid. These findings suggest a potential role for a specific IM community and functional profile in the pathogenesis of NAFLD.

## Introduction

Nonalcoholic fatty liver disease (NAFLD) is the most prevalent chronic liver disease in the Western world^1^ and it is closely associated with obesity, insulin resistance (IR) and diabetes, dyslipidemia, and coronary artery disease^2^, all of which are manifestations of the metabolic syndrome.

The pathogenesis of NAFLD is complex. Research suggests that factors such as genetics^3^, lipid peroxidation^4^, IR associated with obesity^5^, and diet^6^ may contribute. In addition, emerging research suggests a role for the intestinal microbiome (IM) where dysbiosis and bacterial metabolism and products^7^ may influence NAFLD pathogenesis through effects on nutrient digestion and absorption, appetite regulation, host gene expression, and immune function^8^.

Recent human studies have shown associations between IM composition and NAFLD^9–16^, but whether these associations were due to the presence of NAFLD itself or other factors associated with NAFLD, such as increased body mass index (BMI) and IR, was not clear. Most studies did not even consider these factors^10,12–14^. In addition, some of these studies did not perform liver biopsies in either the patient group^10,12,16^ or controls^10–14,16^, or did not assess other factors that may affect IM such as nutritional intake, environment, or physical activity^12–16^. In a previous study^9^ using qPCR, we found an inverse association between the presence of NASH and percentage of Bacteroidetes in the stool, and this was independent of dietary intake and BMI. However, the method only allowed for the assessment of a very limited number of taxa. Also, bacterial products were not analyzed and these products may play a role in the relationship between NAFLD and IM^17^.

The goal of the present study was to determine if there is an association between dysbiosis and NAFLD independently of obesity and IR, by using advanced sequencing technology to characterize IM, in patients with biopsy-proven NAFLD and healthy living liver donors as healthy controls (HC). To ensure robustness of our conclusions to statistical assumptions, the differences in taxa abundances were assessed by two different methods. We additionally quantified select microbial taxa found to be differentially abundant in next generation sequencing data using qPCR, and examined their relationship with NAFLD, BMI and IR. In addition, we measured serum and fecal metabolites using nuclear magnetic resonance spectrometry.

## Methods

### Subjects

In this prospective cross-sectional study, adult participants were recruited at the University Health Network (UHN), Toronto, Ontario, Canada. For NAFLD, subjects with persistently elevated liver enzymes were assessed by hepatologists at UHN and NAFLD was confirmed using standard medical practice to rule out other liver conditions. Patients who accepted to have a liver biopsy were then referred for the study. Inclusion criteria were: age >18 years and biopsy-confirmed NAFLD. Exclusion criteria for patients were: liver disease other than NAFLD, anticipated need for liver transplantation within a year or complications of end-stage liver disease such as variceal bleeding or ascites; concurrent medical illnesses; and contraindications for liver biopsy.

**Figure 1:**
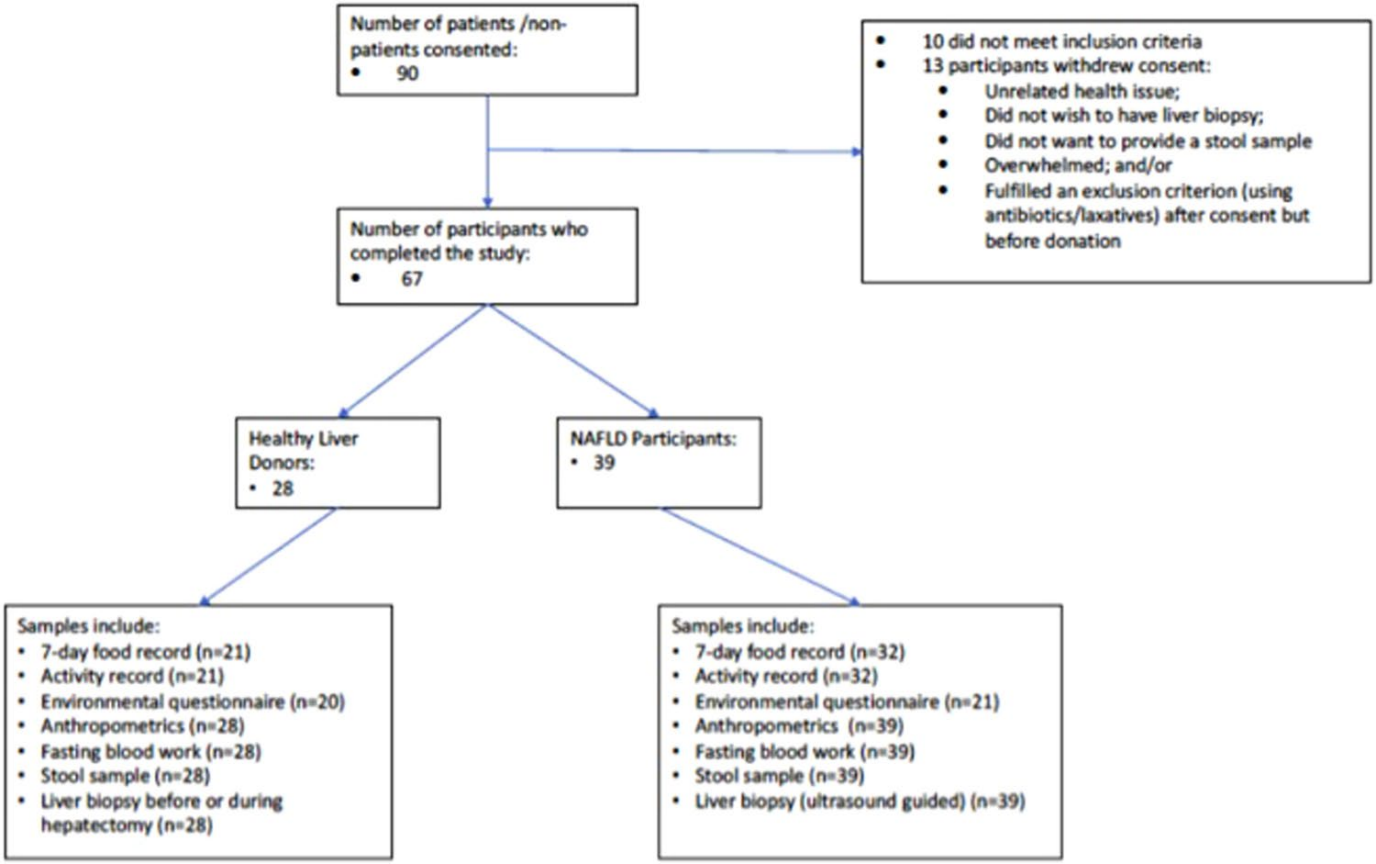
Subject recruitment and sample collection flow chart.

Healthy liver donors from the living donor transplant program served as healthy controls (HC) and as per program protocol a healthy liver was confirmed either by biopsy, computed tomography, and/or medical resonance imaging before partial hepatectomy. For HC the exclusion criteria were: any medical reason excluding them from the live liver donation as per program protocol. Other exclusion criteria for all subjects were: use of medications known to cause or exacerbate steatohepatitis or antibiotics, pre- or probiotics in the preceding 6 months; consumption of more than 20 g of alcohol/day; use of vitamin E or fish oil supplements; chronic gastrointestinal diseases, previous gastrointestinal surgery modifying the anatomy; pregnancy or lactating state. Figure 1 provides an overview of subject recruitment and study completion.

This study was registered with ClinicalTrials.gov (NCT02148471). Additional trial outcomes are reported in previous publications^18,19^. A manuscript reporting on immune response is pending submission.

### Ethics

The study protocol was approved by the UHN and University of Toronto Research Ethics Boards and conformed to the ethical guidelines of the 1975 Declaration of Helsinki. All participants gave their informed written consent. All methods were performed in accordance with the relevant guidelines and regulations of UHN and the University of Toronto.

### Measurements

Patients provided one stool sample and one fasting blood sample, underwent anthropometric measurements (height, weight, BMI) and completed a 7-day food record, a 7-day activity log and an environmental questionnaire before the liver biopsy. Healthy adults undergoing assessment for living liver donation were approached during their first screening appointment. Upon consent, subjects completed the same tests. Histology was obtained during a pre-donation biopsy (performed to verify the healthy state of the liver) or during hepatectomy. The study participants’ demographics, smoking, alcohol consumption history, medications and supplement use were reviewed and collected.

The food record included all food and beverages consumed over seven days using the 2D Food Portion Visual Chart (Nutrition Consulting Enterprises, Framingham, MA) to estimate portion sizes. Macro- and micronutrient intakes were calculated using Food Processor Diet and Nutrition Analysis Software (Version 7, ESHA Research, Salem, OR).

Physical activity logs were recorded for seven days concurrent with the food records. Participants were asked to list any activity, duration, and intensity level (mild, moderate, strenuous, and very strenuous). Daily physical activity units were calculated: 1 unit = 30 minutes mild, 20 minutes moderate, 10 minutes strenuous, or 5 minutes very strenuous activity^20^.

Liver biopsies were taken percutaneously (needle biopsy) for NAFLD patients and intra-operatively (wedge biopsy) for HC. In some HC a pre-donation needle biopsy was used. A portion of each biopsy was fixed in 10% formalin, embedded in paraffin wax, sectioned, and stained with hematoxylin and eosin for morphologic evaluation and Prussian Blue stain to rule out iron loading. Steatosis, inflammation, and fibrosis were assessed using the Brunt system^21^. Disease severity was additionally evaluated using the NAFLD Activity Score (NAS)^22^. The pathologist was blinded to the study.

Blood was drawn in a fasting state. Measures of glucose metabolism included plasma glucose, insulin, hemoglobin A1c (HbA1c), and homeostasis model assessment estimated IR (HOMA-IR)^23^. Liver enzymes and lipid profile (total cholesterol, low density lipoprotein (LDL), high density lipoprotein (HDL) and triglycerides (TG)) were measured at the UHN Laboratory Medicine Program using the Architect c8000 system (Abbott Laboratories, Abbott Park, Illinois). LDL was calculated from total cholesterol – HDL. Fasting plasma glucose and plasma insulin were measured by the enzymatic hexokinase method and radioimmunoassay, respectively^24^.

Each patient and HC collected one stool sample, which was frozen immediately in their home freezer (−20 °C). Within 24 hours, they brought the frozen sample in an insulated bag with cooling elements to UHN, where it was stored at −80 degree centigrade. Subsequently, stool samples were transferred into a sterile masticator bag, thawed and homogenized using a masticator blender (IUL, S.A., Barcelona, Spain). The samples were aliquoted, immediately placed on dry ice, and then transferred to −80 °C for storage until DNA extraction and metabolite analysis.

For IM analysis, bacterial DNA was extracted using the E.Z.N.A.^TM^ Stool DNA Isolation Kit (Omega Bio-Tek, Doraville, GA, USA) as previously described^9^. Fecal DNA samples were stored at −80 °C until being amplified by PCR using the Earth Microbiome V4 primer set^25^, with the addition of combinatorial in-line barcodes so that all the samples could be sequenced in the same sequencing run^26^. The amplified DNA was then sequenced on the Illumina MiSeq platform with paired end 220 nucleotide reads, producing 25 million reads in total. Reads were overlapped with PANDAseq assembler^27^, clustered into Operational Taxonomic Units (OTUs) using UCLUST^28^, and annotated with the SILVA database^29^ using mothur^30^, producing a table of counts per OTU per sample. 7.5 million of the reads were successfully overlapped and annotated into 532 OTUs. A generalized workflow for processing 16S rRNA gene sequencing reads is available at https://github.com/ggloor/miseq_bin. Two sequencing runs were conducted with the results combined into one data matrix which was used for the analysis. The resulting data matrix was filtered at 1% OTU relative abundance (OTUs had to be at least 1% abundant in at least 1 sample) and the remaining OTUs (161) were included into the analysis. The lowest read count per sample after OTU filtering was 15,790, and the highest read count per sample was 210,867. The sequencing and pre-processing data for analysis was performed at the Microbiota Profiling Service, Department of Biochemistry, University of Western Ontario, London, Ontario, Canada.

To further evaluate the sequencing data, the abundance of selected taxa was estimated in fecal samples using qPCR. Primers and probe sequences used in this study are listed in Supplementary Table 2. These included: total bacteria, Bacteroidetes, *Alistipes, Coprococcus, Ruminococcus, Lactobacillus* and *F. prausnitzii*. The runs were performed in triplicates using 50 ng of fecal DNA in the 7900HT thermocycler (Applied Biosystems, Foster City, CA) as previously described^9^. The number of cells for each bacterial group in fecal samples was calculated from standard curves and expressed as colony forming unit per gram (CFU/g) of wet feces, normalized to total counts (relative abundance).

Serum metabolites, including ethanol, were measured at the Metabolomic Innovation Centre (University of Alberta, Edmonton, AB, Canada) using nuclear magnetic resonance (NMR) spectrometry on a 500 MHz Inova (Varian Inc., Palo Alto, CA) spectrometer (Varian Inc., Palo Alto, CA). For stools, NMR spectroscopy was also used from the same center to identify eight metabolites of interest (acetic acid, butyric acid, formic acid, isobutyric acid, isovalerate, L-lactic acid, propionate, succinate). For the measurement, fecal samples were prepared by mixing 20 mg of frozen fecal material with 1 mL of saline phosphate buffer in deuterium oxide, followed by centrifugation (18,000 × g, 1 min). Fecal supernatants were filtered through 0.2 μm membrane filters^31^.

### Statistical Analysis

Descriptive summaries of all measurements were calculated: means and standard deviations (SD) for normal variables, medians and first and third quartiles for skewed variables, and proportions for categorical variables. Plots were used as appropriate to examine the data. Bivariate relationships were assessed using Pearson’s or Spearman’s correlation coefficients, Chi-square and Fisher exact tests where appropriate. Comparisons across diagnosis groups were performed using one-way ANOVA followed by the t-test (normally distributed data), data were log-transformed to normalize distribution if necessary, or using the Kruskal-Wallis test followed by Wilcoxon rank-sums test (not normally distributed data). All tests were two-sided and performed at the 5% significance (alpha) level. Bonferroni’s correction method was used to account for multiple comparisons between diagnostic groups.

For next generation sequencing, differences in overall IM characteristics of diagnostic groups were assessed in QIIME^32^. Principal coordinate analysis based on weighted UniFrac distance matrix^33^ was performed to visualize and examine differences in overall microbial community compositions among the three groups. For differential abundance analysis read counts were quantile normalized^34^. In order to ensure robustness of our conclusions to statistical assumptions we applied 3 different analysis methods and made inferences if the results of all analyses were consistent. First, zero-inflated Gaussian models to perform 3-group comparisons^35^. Then pairwise differences between groups were assessed by two non-parametric methods: Metastats^35,36^ and Wilcoxon rank sum test (details in Supplemental Materials). Correction for multiple comparisons was done using Benjamini-Hochberg false discovery rate^37^, and q-value < 0.05 was considered statistically significant. In some cases, following a recent publication^15^ we were less stringent in controlling for multiple comparisons, and considered higher q-value thresholds (but less than 0.1).

For qPCR data, due to non-normality and heteroscedasticity of distribution for most groups of bacteria, we used non-parametric methods: Kruskal-Wallis and Wilcoxon rank sum tests for between group comparisons, and Spearman correlation coefficients and partial Spearman correlation coefficients to examine relationship between relative abundances and other variables (diagnosis (treated as ordinal variable), BMI, IR, metabolites).

Analysis was performed using tools SAS 9.4, R 3.2.5 and QIIME software.

### Data Availability

The datasets generated during and analysed during the current study are not publicly available due to privacy restrictions but some blinded data may be made available from the corresponding author on reasonable request.

## Results

### Patient characteristics

The study included 15 patients with biopsy-proven simple steatosis (SS), 24 with nonalcoholic steatohepatitis (NASH) and 28 healthy liver donors from the living donor transplant program as HC. Patient demography and clinical characteristics are presented in Table 1. The HC group was younger compared to both SS and NASH with significantly lower median BMI compared to NASH. There were no significant differences in gender distribution. As expected, liver transaminases were progressively increased from HC to SS to NASH. Four NASH and 1 SS patients who were taking anti-diabetic drugs (none were on insulin therapy) as well as 3 patients with HOMA-IR >15 (possibly a consequence of non-fasting before the blood test), were excluded from analyses related to diabetes parameters (HbA1c, HOMA-IR). SS and NASH patients had significantly higher HbA1C compared to HC, and NASH had higher HOMA-IR compared to both SS and HC.

**Table 1:** Characteristics of patients included in the study.

	HC (n = 28)^*^ n (%)	SS (n = 15)^*^ n (%)	NASH (n = 24)^*^ n (%)	p-value**
Gender, male	15 (54%)	9 (60%)	11 (46%)	0.7
Ethnicity, Caucasian (n = 41)	16 (80%)	4 (44%)	7 (58%)	0.15
	**Median (min, max)**	**Median (min, max)**	**Median (min, max)**	
Age, yrs	36.5_A,B_ (21, 58)	48_B_ (33, 61)	46.5_A_ (29, 68)	***0.0003***
BMI, kg/m^2^	26.6_A_ (19.5, 35.3)	27.4 (23.5, 44.2)	32.1_A_ (24.17, 49.53)	<***0.0001***
AST, U/L	19.5_B,C_ (12, 29)	26_A,C_ (16, 53)	45_A,B_ (18, 114)	<***0.0001***
ALT, U/L	17.5_B,C_ (7, 41)	45_A,C_ (14, 116)	70_A,B_ (22, 168)	<***0.0001***
HbA1c*** (n = 62)	0.05_A,B_ (0.04, 0.07)	0.06_B_ (0.05, 0.09)	0.06_A_ (0.05, 0.07)	***0.0003***
HOMA-IR*** (n = 48)	1.16_A_ (0.53, 7.59)	1.12_B_ (0.54, 6.83)	3.27_A,B_ (1.21, 14.54)	***0.0006***
NAFLD Activity Score (n = 53)	0	1 (1, 4)	4.5 (3, 8)	<***0.0001***

*For some variables numbers were lower due to missing values/exclusions.

**Kruskal Wallis test or Chi-square test as appropriate.

***Patients on diabetes drugs excluded.

Same subscript letters denotes significant differences.

HC: healthy controls, SS: simple steatosis, NASH: nonalcoholic steatohepatitis, AST: aspartate transaminase, ALT: alanine transaminase, HbA1c: Hemoglobin A1c, HOMA-IR: homeostasis model assessment estimated insulin resistance.

For the liver histology, NAFLD Activity Score for SS patients ranged from 1 to 4 (median 1), and for NASH patients from 3 to 8 (median 4.5). Details on liver histology (Supplementary Table 3), level of activity (Supplementary Table 4), environmental questionnaire (Supplementary Table 5), and diet (Supplementary Tables 6 and 7) are reported in the supplementary section. Overall, there were no significant differences in level of activity and diet composition. There was a higher proportion of HC born in Canada but ethnicity was similar between groups for those who filled out the questionnaire.

### Intestinal Microbiome

#### Next generation sequencing data

161 OTUs passed a 1% filter and were retained for analysis. The filtered OTUs were aggregated (after normalization) into higher taxonomic levels: 8 phyla, 25 families and 44 genera.

The analysis of overall community structure and composition, including Simpson diversity metric and principal coordinate analysis did not reveal differences between the groups. Simpson diversity was not significantly different between the 3 groups (p-value 0.5) (Supplementary Figure 1). There was no clear separation between groups in weighted UniFrac principal coordinate analysis plot (Supplementary Figure 2).

To investigate differences between diagnostic groups in differential abundance of individual taxa, we first fit ANOVA ZIG model comparing 3 groups on OTU and genus levels, followed by pairwise comparisons between each diagnostic category (Supplementary Data). Size and direction of differences for HC vs SS and HC vs NASH comparisons were similar in both OTU and genus levels, and differentially abundant groups were similar in both of these comparisons. At the same time, the differences between SS and NASH groups were never significant and size of differences was small, suggesting similar microbiome characteristics for NASH and SS in our study. Based on this and taking into account low sample size in SS group, we combined NASH and SS into one NAFLD group for further analysis.

**Figure 2:**
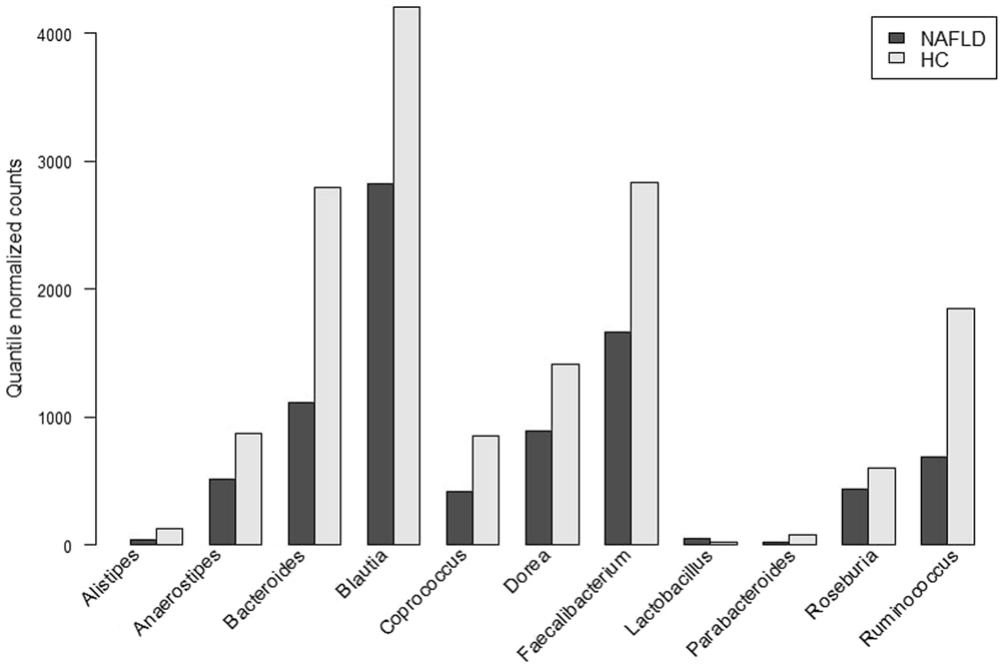
Medians of differentially abundant genera based on 16S sequencing data.

Based on the results of Metastats and Wilcoxon tests we identified 7 OTUs, 10 genera (for 4 of those q-value in Wilcoxon test did not reach 0.05 threshold, but was <0.1), and 5 families as less abundant in NAFLD than in HC. One OTU, 1 genus (*Lactobacillus*), and 1 family (*Lactobacillaceae*) were more abundant in NAFLD. Two dominant phyla, Bacteroidetes and Firmicutes were less abundant in NAFLD (Table 2). Differentially abundant genera are further summarized in Fig. 2.

**Table 2:** Taxa identified as differentially abundant in patients with NAFLD (SS or NASH) compared to HC by Metastats and Wilcoxon rank sum test.

*Taxa*	Metastats					Wilcoxon test		Quantile normalized counts	
								median (min, max)	
	log fold change^1^	p-value min^2^	p-value max^2^	q-value min^2^	q-value max^2^	p-value	q-value	HC	NAFLD
								n = 28	n = 39
***OTU level***									
*Firmicutes;Clostridia;Clostridiales; Lachnospiraceae;Anaerostipes;11*	−0.761	0.0002	0.0007	0.008	0.028	0.0003	0.017	873.8	513.2
								(412.1, 3191.3)	(181.8, 2543.3)
*Firmicutes;Clostridia;Clostridiales; Lachnospiraceae;Incertae_Sedis; 13*	−0.681	0.002	0.003	0.039	0.06	0.001	0.021	636.6	399.4
								(142.5, 3652.8)	(183.2, 2090)
*Firmicutes;Clostridia;Clostridiales; Ruminococcaceae;Faecalibacterium; 2*	−0.751	0.002	0.002	0.039	0.046	0.001	0.029	1670.9	946.2
								(308.6, 8565.5)	(375.5, 5075.3)
*Firmicutes;Clostridia;Clostridiales; Lachnospiraceae;unclassified; 20*	−0.577	0.001	0.002	0.039	0.046	0.001	0.025	434.3	277.3
								(133.9, 3712.2)	(159.3, 831.9)
*Bacteroidetes;Bacteroidia;Bacteroidales; Bacteroidaceae;Bacteroides; 23*	−0.989	0	0.0001	0	0.016	0.0004	0.017	492.4	194.4
								(128.7, 1768.7)	(95.9, 882.5)
*Firmicutes;Clostridia;Clostridiales; Lachnospiraceae;Dorea; 31*	−0.77	0.0002	0.0003	0.008	0.016	0.0004	0.017	267.6	140.5
								(86.6, 1280.1)	(64.3, 503.7)
*Firmicutes;Clostridia;Clostridiales; Lachnospiraceae;Blautia; 6*	−0.685	0	0.0002	0	0.016	0.0004	0.017	1182.7	657.1
								(398.9, 3296.7)	(338.4, 1959)
*Firmicutes;Bacilli;Lactobacillales; Lactobacillaceae;Lactobacillus; 84*	1.258	0.007	0.008	0.076	0.078	0.001	0.019	9.8	23.8
								(2.1, 740)	(1.6, 701.6)
***Genus level***									
*Alistipes*	−1.307	0.0003	0.001	0.004	0.005	0.001	0.005	126.8	38.9
								(21.8, 1779.6)	(18.5, 696)
*Anaerostipes*	−0.761	0.001	0.001	0.005	0.009	0.0003	0.005	873.8	513.2
								(412.1, 3191.3)	(181.8, 2543.3)
*Bacteroides*	−0.874	0.0004	0.001	0.004	0.006	0.0004	0.005	2798.4	1117.2
								(654.2, 6083.6)	(425.9, 7385.7)
*Blautia*	−0.48	0.002	0.003	0.014	0.021	0.003	0.016	4205.6	2823.6
								(2040, 8236)	(1154, 10964)
*Coprococcus*	−0.778	0.005	0.006	0.027	0.03	0.014	**0.06**	850.5	413.6
								(174.6, 2877.3)	(151.1, 1848.1)
*Dorea*	−0.596	0.004	0.006	0.027	0.031	0.01	0.048	1414.4	890
								(574.1, 6919.3)	(425.7, 4320.7)
*Faecalibacterium*	−0.599	0.006	0.006	0.027	0.031	0.003	0.017	2837.1	1663.1
								(552.3, 8886.2)	(699.4, 8785.9)
*Lactobacillus*	1.232	0.013	0.016	0.046	0.05	0.001	0.005	20.7	54.6
								(2.9, 5067.1)	(9.2, 720.9)
*Parabacteroides*	−1.045	0.012	0.012	0.046	0.047	0.017	**0.068**	76.6	24.5
								(7, 438.9)	(4.7, 423.6)
*Roseburia*	−0.426	0.015	0.016	0.047	0.05	0.021	**0.071**	600	432.9
								(266.7, 1926.5)	(218.7, 1412.6)
*Ruminococcus*	−0.7	0.008	0.011	0.035	0.047	0.019	**0.07**	1846.1	692.3
								(323.5, 4628)	(347.5, 3160.2)
***Family level***									
*Bacteroidaceae*	−0.874	0.0004	0.001	0.003	0.004	0.0004	0.003	2798.4	1117.2
								(654.2, 6083.6)	(425.9, 7385.7)
*Lachnospiraceae*	−0.563	0.0001	0.0002	0.001	0.003	0.0001	0.002	16822.1	12328
								(9212, 34155)	(6477, 26139)
*Lactobacillaceae*	1.232	0.012	0.013	0.043	0.046	0.001	0.004	20.7	54.6
								(2.9, 5067.1)	(9.2, 720.9)
*Porphyromonadaceae*	−1.413	0.001	0.001	0.003	0.004	0.001	0.005	163.8	37.3
								(9.3, 7343.7)	(10.1, 463.1)
*Rikenellaceae*	−1.307	0.0001	0.001	0.001	0.004	0.001	0.004	126.8	38.9
								(21.8, 1779.6)	(18.5, 696)
*Ruminococcaceae*	−0.774	0	0.0001	0	0.001	0	0.001	8298.6	4881.6
								(3328, 17756)	(2139, 14877)
***Phylum level***									
*Bacteroidetes*	−0.763	0.003	0.003	0.012	0.013	0.003	0.011	3744	1497.7
								(787.4, 12620.1)	(613.1, 10942.2)
*Firmicutes*	−0.622	0	0	0	0	0	0.0003	30975.9	21209.9
								(19486, 57571)	(10571, 46704)

^1^Negative parameter denotes that taxon is more abundant in HC, positive – that it is more abundant in NAFLD.

^2^Estimated in 3 rounds of permutations, 10,000 instances each.

HC: healthy controls, SS: simple steatosis, NAFLD: nonalcoholic fatty liver disease, NASH: nonalcoholic steatohepatitis.

#### qPCR Data

Based on 16S data analysis results and similar taxa previously reported^11,12,14,15^, the following taxa were selected to be quantified by qPCR: Bacteroidetes which included *Parabacteroides* and *Bacteroides* genera found on sequencing as well as *F. prausnitzii*, *Alistipes, Coprococcus, Ruminococcus and Lactobacillus*. The relative abundance of the targeted bacterial groups is presented in Table 3.

**Table 3:** Relative abundances of bacterial taxa quantified by quantitative PCR.

	HC		SS		NASH*		p-value**
	n=28		n=15		n=24		
	Median	(min, max)	Median	(min, max)	Median	(min, max)	
Bacteroidetes	2.4	(0.1, 30.5)	3.5	(0.1, 10.8)	0.78	(0, 13.3)	0.19
*Alistipes*	1	(0, 5.1)	0.2	(0, 4.6)	0.6	(0, 5.9)	0.14
*Coprococcus*	0.4_A, B_	(0, 15.4)	0.0_A_	(0, 2.6)	0.0_B_	(0, 1.2)	***0.005***
*Lactobacillus*	0	(0, 0.4)	0	(0, 0.6)	0	(0, 0.3)	0.7
*Ruminococcus*	13.2_A, B_	(7.9, 27.4)	2.6_A_	(0.3, 16.4)	2.0_B_	(0, 9.6)	***<0.0001***
*Faecalibacterium prausnitzii*	11.7_A, B_	(0.4, 22.1)	2.2_A_	(0, 10.2)	1.7_B_	(0, 12.4)	***<0.0001***

Abundances were calculated as ratio of colony forming units (CFU) to total bacteria CFU in the sample. Same subscript denotes significant difference (after Bonferroni correction).

HC: Healthy controls, SS: simple steatosis, NASH: nonalcoholic steatohepatitis.

*For 1 NASH patient there was not enough DNA for Coprococcus, Alistipes and F. prausnitzii, so the sample size was reduced to n = 23.

**Kruskal Wallis test. Same subscript letters denote significant differences.

The following groups were less abundant in NASH and SS patients compared to HC: *Ruminococcus, F. prausnitzii* and *Coprococcus* (Table 3). Even though the median abundance for all taxa except *Coprococcus* were lower in NASH than in SS, the differences between the two patient groups did not reach statistical significance. There were no statistically significant differences between diagnostic groups for Bacteroidetes, *Alistipes* and *Lactobacillus*.

**Figure 3:**
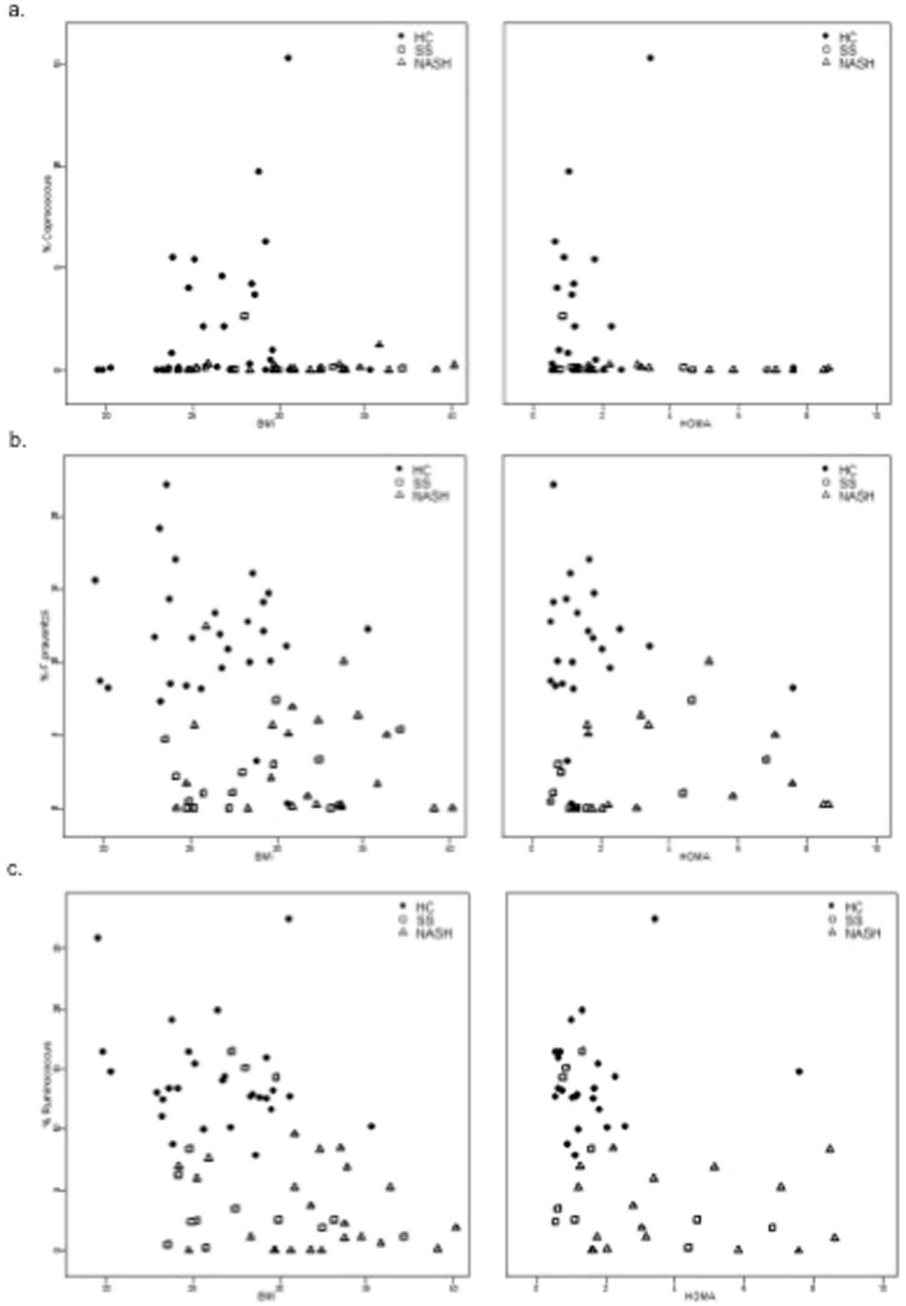
Scatterplots illustrating relationship between relative abundance of relevant bacterial taxa by qPCR, diagnosis and body mass index (left panel), and homeostasis model assessment estimated insulin resistance (HOMA-IR, right panel) (**a**) *Coprococcus*; (**b**) *F. prauznitzii* (**c**) *Ruminococcus*.

To investigate whether the association between taxa and NAFLD was due to confounding factors like BMI and IR, we calculated two sets of partial Spearman correlations: (1) between these factors and bacterial abundances, controlling for diagnosis and; (2) between bacterial abundances and diagnosis controlling for BMI and HOMA-IR (Table 4). For the taxa that differed between HC and NAFLD, partial correlations between diagnosis status and abundance were highly significant after controlling for BMI or IR. After controlling for diagnosis, there was no evidence of relationship between BMI and bacterial abundances, and for HOMA-IR only weak evidence of a positive relationship with *F. prausnitzii* (Fig. 3). Therefore, our analysis suggests that NALFD is associated with reduced abundance of *Ruminococcus, Coprococcus* and *F. prausnitzii* independent of BMI and IR.

**Table 4:** Partial Spearman correlation coefficients (p-value); diagnosis was treated as ordinal variable (1-healthy control, 2-simple steatosis, 3-nonalcoholic steatohepatitis).

	BMI (controlling for diagnosis)	HOMA-IR (controlling for diagnosis)	diagnosis (controlling for BMI)	diagnosis (controlling for HOMA IR)
%*Coprococcus*	0.18 (0.1)	−0.11 (0.5)	***−0.40*** (***0.0009*)**	***−0.40*** (***0.006*)**
%*Faecalibacterium prausnitzii*	0.02 (0.9)	0.28 (0.057)	***−0.61*** (<***0.0001*)**	***−0.68*** (<***0.0001*)**
%*Ruminococcus*	−0.09 (0.5)	−0.04 (0.8)	***−0.69*** (<***0.0001*)**	***−0.70*** (<***0.0001*)**

HOMA-IR: homeostasis model assessment estimated insulin resistance.

We then tested whether there was a relationship between liver histology (% steatosis, lobular inflammation, ballooning, fibrosis, NAFLD activity score, as seen in Supplementary Table 3) and relative abundances of quantified taxa within the NAFLD group (NASH+SS, n = 38). The results were negative except for a weak negative relationship between *Ruminococcus* and lobular inflammation (ρ = −0.33, p-value 0.04) (Supplementary Table 8). Considering that these p-values were not controlled for multiple comparisons, this result can be due to Type 1 error.

#### Bacterial Products

NAFLD patients had significantly higher concentrations of fecal propionate and isobutyric acid as well as serum 2-hydroxybutyrate and L-lactic acid (Table 5). The ratio of the short-chain fatty acids (SCFA) acetate:propionate:butyrate in our HC and NAFLD patients was approximately 62:23:15 and 61.5:22.5:16, respectively, which is similar to commonly quoted ‘normal’ ratios of 60:20:20 and 60:25:15^38^. Serum ethanol was not different between groups. The abundance of *Ruminococcus, F. prausnitzii* and *Coprococcus* was not correlated with the concentration of the measured bacterial products (Supplementary Table 9).

**Table 5:** Bacteria-related fecal and serum metabolites.

Fecal Metabolite (μM)	Healthy Controls		NAFLD Patients		p-value
	n	Median (Q1, Q3)	n	Median (Q1, Q3)	
Butyric Acid	28	1366 (993, 2072)	38	1753 (1130, 2266)	0.2
Propionate	28	2052 (1598, 2419)	38	2485 (1985, 3649)	***0.019***
Acetic Acid	28	5665 (4025, 7061)	38	6781 (4619, 8384)	0.088
Formic Acid	28	60 (50, 73)	38	56 (42, 68)	0.3
Total SCFA	28	9122 (6747, 11757)	38	11366 (8097, 13320)	0.05
(sum of above)					
Isobutyric Acid	28	249 (199, 285)	38	293 (239, 372)	***0.017***
Isovalerate	28	181 (115, 223)	38	172 (136, 208)	0.9
Succinate	28	121 (67, 175)	38	94 (58, 187)	0.5
L-Lactic Acid	28	37 (29, 47)	38	41 (30, 58)	0.3
**Serum Metabolite (μM)**	**Healthy Controls**		**NAFLD Patients**		**p-value**
	**n**	**Median (Q1, Q3)**	**n**	**Median (Q1, Q3)**	
Acetic Acid	26	48 (3767)	33	37 (33, 59)	0.09
Formic Acid	26	55 (47, 63)	33	58 (51, 61)	0.4
2-Hydroxybutyrate	26	48 (39, 66)	33	69 (48, 78)	***0.0096***
Isobutyric Acid	26	0 (0, 0)	33	0 (0, 0)	0.2
L-Lactic Acid	26	1370 (1069, 1807)	33	1958 (1523, 2288)	***0.0056***
Ethanol	26	23.5 (0, 50)	33	0 (0, 41)	0.8
Endotoxin (EU/mL)	24	6.0 (5.4, 6.4)	38	6.1 (5.3, 6.5)	0.7

NAFLD: nonalcoholic fatty liver disease = patients with simple steatosis and those with nonalcoholic steatohepatitis combined, SCFA: short-chain fatty acids.

## Discussion

We demonstrated, in a well-characterized adult population, that NAFLD was associated with reduced abundance of several bacterial taxa (*Ruminococcus, Coprococcus* and *F. prausnitzii*) independent of BMI and IR. Additionally, selected bacterial products related to fermentation (SCFA) were higher in NAFLD patients compared to HC. Our results are consistent with previous studies, comparing NAFLD and HC^11–14^ and investigating the association between IM and NAFLD severity^15^. In all of these studies, comparisons were cross-sectional, except for Wong *et al*. who also compared IM of NASH patients before and after probiotic intervention. All reported evidence of dysbiosis in NAFLD patients when compared to HC. Only one study^14^ used RT-PCR to validate the results of the next-generation sequencing analyses but only with one genus (*Lactobacillus*). A pediatric study^11^ is also the only one that controlled for confounding factors when assessing the association between IM and NAFLD.

The patient groups in this study differed as expected based on the disease state. SS and NASH patients were older and NASH had a significantly higher BMI than HC. Liver enzymes and insulin resistance measures were also higher in the NAFLD groups. All of these findings were expected given the typical NAFLD population^39^. In regard to age, previous studies have identified changes in IM over the lifespan, however, most changes occur in early childhood and elderly adults >70 years old^40–42^. Research into IM changes in the elderly has often been confounded by dietary changes, medication use, and declining overall health. Therefore, a cut-off age for IM change is undeterminable^43^. Our total population, with a maximum age of 68 years is therefore unlikely to have experienced a confounding effect of age on IM. In regard to BMI, the median BMI of the HC was in the overweight range 26.6 (19.5, 35.3) [median (min, max)]. However, the groups were characterized by liver histology, and HC were confirmed to have a normal liver. This is a strength in our study as, to our knowledge, no other studies published on NAFLD had a HC group with confirmed normal liver biopsies. Another strength is the HC group with a median BMI in the overweight range. This BMI range in the HC group is reflective of the current Canadian population^44^. The smaller difference in BMI between NAFLD and HC reduces potential confounding effects on IM when comparing the groups.

The lower abundance of *Coprococcus* or *Faecalibacterium* in NAFLD was consistent with other studies^11,13,14^. *Ruminococcus* was previously reported only by Zhu *et al*., but the family Ruminococcaceae was consistently reported as less abundant in NAFLD^12,14^. In our study, liver biopsy was used to diagnose NAFLD while some of these previous studies^12,14^ relied mostly on imaging. Also, their healthy controls had no liver biopsy, were much leaner compared to NAFLD^12–14^ and differences in BMI were not considered in the analysis. This could have influenced the results.

Our analyses did not show any association between IM and NAFLD severity or correlation between specific taxa and histological parameters. This is contrary to the findings by Boursier *et al*.^15^, who reported higher abundance of *Bacteroides* in NASH compared to SS patients and a positive association between *Ruminococcus* and fibrosis severity, independent of metabolic factors (assessed only by the presence or absence of diabetes). The diverging results can have multiple explanations. First, there can be differences in disease severity in study samples: our patients had milder disease (NAFLD activity score median 1 for SS and 4.5 for NASH) and only 18% had severe fibrosis or cirrhosis (stage 3 or 4). Secondly, differences in patient populations, environmental factors and dietary habits in Canada vs Europe, could have influenced associations between IM and disease severity. Finally, in our study, more stringent criteria to select differentially abundant species by controlling for multiple comparisons were applied compared to Boursier’s study, which did not correct for increased chances of type I error when testing the hypothesis of differential abundance for multiple taxa.

Although the sequencing data showed that the Bacteroidetes phylum and one OTU within the phylum were lower in NAFLD versus HC, this was not confirmed by qPCR. Using qPCR, we previously showed^9^ a lower percentage of Bacteroidetes in NASH versus HC and SS. However, it is possible that since the qPCR primers targeted all Bacteroidetes, we may have missed sub-phylum differences if the differences were less pronounced in this study compared with results from Mouzaki *et al*.^9^. Others also reported conflicting results for this taxon. Boursier *et al*. found higher proportion of Bacteroidetes in NASH compared to SS^15^, Zhu *et al*. also found a higher proportion in pediatric NASH patients and those with obesity compared to lean controls^11^, whereas others^12,14^ did not mention it among differentially abundant taxa in NAFLD.

We found lower abundance of *F. prausnitzii, Coprococcus* and *Ruminococcus* in NAFLD patients. *F. prausnitzii* is an anti-inflammatory commensal bacterium and its abundance is reduced in inflammatory bowel disease^45^ and metabolic syndrome^46^. *Coprococcus* was also lower in NAFLD patients, similar to the report by Zhu *et al*.^11^. A lower abundance of *Coprococcus* has also been observed in other inflammatory conditions^47^. Therefore, it is conceivable that lower abundance can promote chronic inflammation that may contribute to NAFLD pathogenesis. *Ruminococcus* belong to the family Ruminococcaceae which are also fermenting anaerobes that lead to the production of SCFA^48^. Both Zhu *et al*.^11^ and Raman *et al*.^12^ found a lower abundance of Ruminococcaceae in NAFLD patients, however, not in this particular genus.

We expected to see differences in SCFA between groups. However, in feces, only propionate and isobutyric acid were higher in NAFLD compared to HC, whereas concentrations of butyrate, acetate, formate, and total SCFA were not different. There were no correlations between SCFA and bacterial abundances. The ratios of acetate:propionate:butyrate were similar to the normal values in both groups^38^. However, the amount and type of products can vary depending on species. For example, *Ruminococcus* genus contains species and strains which can be metabolically versatile^49–51^ which means the amount and type of fermentation products vary according to species. Some can use different substrates like mucus or cellulose, resulting in production of acetate, ethanol, succinate, lactate and formate, but very little butyrate as end products of glucose metabolism^51^. We also found higher serum 2-hydroxybutyrate and L-lactic acid. However, it is difficult to compare our results to previous studies as human data on SCFA in NAFLD are scarce. Wong *et al*. found that NASH patients had a lower prevalence of butyrate producing *Faecalibacterium* (similar to our study), but they did not measure butyrate levels^13^. Raman *et al*.^12^, comparing obese NAFLD diagnosed on imaging versus lean HC subjects, measured fecal volatile compounds and also showed higher levels of SCFA with elevated fecal propionic, butyric and acetic acids in NAFLD. Similarly increased volatile compounds were observed in NAFLD patients^16^ and higher total fecal SCFA, particularly propionate, was also found in overweight and obese adults compared to lean controls^52^. Higher SCFA from bacterial fermentation can lead to increased energy absorption of up to 150 kcal per day^53^. It is conceivable that the higher concentration of fecal propionate and isobutyric acid found in our NAFLD group reflect this phenomenon. Mechanisms other than IM could have also contributed to the higher level of SCFA in NAFLD versus HC, such as slow transit time^38^ reported in obesity^54^ which may have increased fermentation, or differences in absorption rate^55^. 2-hydroxybutyrate (alpha-hydroxybutyrate) is a metabolite formed during amino acid catabolism and glutathione anabolism^56^. Although this compound has been mentioned as an intermediary in the bacterial metabolism of amino acids, it is not clear how much is actually produced and absorbed in the human colon and absorbed into the blood^57^. Oxidative stress in the liver that can be triggered by IR may have contributed to higher 2-hydroxybutyrate level by increasing the production of hepatic glutathione^56,58^.

The strengths of our study were the precise characterization of HC and NAFLD patients through liver biopsy, and the documentation of dietary intake, physical activity, and environmental factors. We applied robust statistical methodology appropriate for analysis of this type of data, including normalization methods and statistical testing. Particularly, we used 2 types of statistical tests for sequencing data to ensure robustness of conclusions. We also applied multiple comparisons corrections whenever appropriate to avoid identifying false positive taxa and confirmed the results with qPCR. The limitation of our study is the relatively low sample size that does not allow for more multivariate analyses and may have prevented us from showing differences between IM of SS and NASH. However, the sample size was similar to other studies^11,13^ that used liver biopsy to characterize NAFLD. The expanding study of the IM discovered numerous environmental factors that may influence the IM. Vitamin D deficiency, for instance, has recently been linked to dysbiosis and NAFLD^59^. In this study, vitamin D status was not examined, but the patient population was from a relatively small geographic region, and vitamin D intake did not differ between groups. Also, we cannot confirm causality owing to the observational design.

In summary, NAFLD had lower abundance of *Ruminococcus, F. prausnitzii* and *Coprococcus* independent of BMI and IR, and higher concentrations of select fecal and serum metabolites, which may suggest a specific IM community and functional profile in these patients. Future metagenomic research would allow for better characterization of this functional profile.

## Electronic supplementary material

Supplementary Materials

Dataset 1
