# Supplementary material for: Nonalcoholic fatty liver disease is associated with dysbiosis independent of body mass index and insulin resistance
Source: Sci Rep. 2018 Jan 23;8:1466. doi: 10.1038/s41598-018-19753-9 (PMC5780381; doi:10.1038/s41598-018-19753-9)
Supplement: Supplementary file 1 — Supplementary Materials [file 41598_2018_19753_MOESM1_ESM.pdf]

Nonalcoholic fatty liver disease is associated with dysbiosis independent of body mass index and insulin resistance

Hannah E. Da Silva (hannah.dasilva@sunnybrook.ca)[1], Anastasia Teterina (anastasia.teterina@gmail.com)[2], Elena M. Comelli (elena.comelli@utoronto.ca)[3], Amel Taibi (amel.taibi@utoronto.ca)[3], Bianca M. Arendt (bianca.arendt@gmail.com)[2], Sandra E. Fischer (Dr.Sandra.Fischer@uhn.ca)[2], Wendy Lou (wendy.lou@utoronto.ca)[4], Johane P. Allard (Dr.Johane.Allard@uhn.ca)[2,3]*

[1] Sunnybrook Health Sciences Center, Toronto, Ontario, Canada; [2] Department of Medicine, Toronto General Hospital, University Health Network, University of Toronto, Toronto, Ontario, Canada; [3] Department of Nutritional Sciences, University of Toronto, Toronto, Ontario, Canada; [4] Dalla Lana School of Public Health, University of Toronto, Toronto, Ontario, Canada

[*] Address correspondence to: Johane P. Allard MD, FRCP(C), University Health Network, Toronto General Hospital, 585 University Ave, Suite 9-NU-973, Toronto, Ontario, Canada M5G 2N2. E-mail: Johane.allard@uhn.on.ca

SUPPLEMENTARY MATERIALS

16S data analysis

Zero-inflated Gaussian models were used as implemented in fitZig function in R metagenomeSeq package version 1.12.1. P-values obtained using empirical Bayes method were used for inference [1]. Metastats method was used as implemented in fitLogNormal function in R metagenomeSeq package version 1.12.1 [2,3]. The method uses independent samples t-test on log2-transformed normalized read counts (1 is added to zero count values to allow for log transformation); Effect size is the point estimate of fold-difference in normalized counts. To take into account possible non-normality of data, the p-value is estimated using permutation method. As permutation-based p-values might slightly differ from instance to instance, we based our conclusions on 3 rounds of 10,000 permutations each. Additionally, to check the robustness of our results to statistical assumptions, we compared the normalized counts between groups using Wilcoxon rank sum test. All p-values were corrected for multiple comparisons using Benjamini-Hochberg false discovery rate [4].

Supplementary Table 1. Environnemental Questionnaire

Patient ID ___________ Visit Date ____________

1. What country were you born in? ______________________

2. If you were not born in Canada, when did you immigrate? ________

3. What is your ethnic background? (Check all that apply)

□ African-Canadian □ Asian □ Caucasian

□ First Nation □ Hispanic □ Hispanic-Black

□ Middle Eastern □ Other ____________ (specify)

4. How were you born?
□ Caesarian section (C-section) □ Natural birth □ I don't know

5a. As an infant, were you ever breastfed?

□ Yes □ Never □ I don't know

5b. If "Yes", how long were you breastfed?

□ Less than 6 month □ More than 6 months □ I don't know

6a. Do you have any allergies or food intolerances?

□ Yes □ No □ I don't know

6b. If "Yes", please specify: __________________

7a. Do you have any history of gastro-intestinal diseases (e.g. inflammatory bowel disease, celiac disease, gastric ulcer)?

□ Yes □ No □ I don't know

7b. If "Yes", please specify: ________________

8a. Did you ever have any major abdominal surgery?

□ Yes □ No □ I don't know

8b. If "Yes", please specify: __________________

Date of surgery: ________________ (mm/yyyy)

9a. During the last 6 months, did you use any antibiotics?

□ Yes □ No □ I don't know

9b. If "Yes", when was the last time you used antibiotics?
__________________ (dd/mm/yy)

10a. During the last 6 months, did you use any laxatives?

□ Yes □ No □ I don't know

10b. If "Yes", how often do you use a laxative? ________________

10c. If "Yes", when was the last time you used a laxative?

_______________ (dd/mm/yy)

11. Are you using any pre- or probiotic or synbiotic products on a regular basis (as a food product or as a supplement such as tablets, powder, etc.)?

□ Yes □ No □ I don't know
Name(s) of the product(s): _____________________

12. Did you travel outside of North-America during the last 6 months?

□ Yes Country: ________________ □ No

13a. Did you have any gastrointestinal infections during the last 6 months (e.g. traveller's diarrhea)

□ Yes □ No □ I don't know

13b. If "Yes", when did you have the last infection?
_____________________ (dd/mm/yy)

14. Do you have any pets at home?

□ Yes What animal(s)? ________________ □ No

15a. Female Participants ONLY: Are you using any contraceptives?

□ Yes Specify: ____________________ □ No
15b. When was the first day of your last period?
_________________ (dd/mm/yy) □ I am past my menopause
15c. Usual length of your menstrual cycle? ___ days □ I don't know

Supplementary Table 2. List of primers and probes used in this study

Target organism	Sequence(5' to 3')	Annealing temp (ºC)	Chemistry	Reference
All Bacteria	Forward: CGGTGAATACGTTCCCGG<br>Reverse: TACGGCTACCTTGTTACGACTT<br>Probe: CTTGTACACACCGCCCGTC	60	TaqMan	Furet 2009 (PMID: 19302550)
Alistipes genus	Forward: ATGGGCATGCGTTGTATTAGC<br>Reverse: CGTGTCTCAGTACCAGTGTGG	55	SYBR Green	This study*
Bacteroidetes phylum	Forward: CCTTCGATGGATAGGGGTT<br>Reverse: CACGCTACTTGGCTGGTTCAG<br>Probe: AAGGTCCCCCACATTG	60	TaqMan	Furet 2009 (PMID: 19302550)
Coprococcus genus	Forward: CATCCTGATGACGGTTTCTTAACC<br>Reverse: GTTGCGGGACTTAACCCA	55	SYBR Green	This study*
F. prausnitzii	Forward: TGTAAACTCCTGTTGTTGAGGAAGATAA<br>Reverse: GCGCTCCCTTTACACCCA<br>Probe: CAAGGAAGTGACGGCTAACTACGTGCCAG	60	TaqMan	Lopez-Siles 2014 (PMID: 24713205)
Lactobacillus genus	Forward: TGGATGCCTTGGCACTAGGA<br>Reverse: AAATCTCCGGATCAAAGCTTACTTAT<br>Probe: TATTAGTTCCGTCCTTCATC	60	TaqMan	Haarman 2006 (PMID: 16597930)
Ruminococcus genus	Forward: GAGTGAAGTAGAGGTAAGCGGAATTC<br>Reverse: GCCGTACTCCCCAGGTGG	60	SYBR Green	Weimer 2008 (PMID: 18535825)

* The 106 bp and 109 bp qPCR primers were developed to target the variable V3 region of the 16S rRNA gene of *Alistipes* and *Coprococcus* respectively. The primers were designed from multiple alignments of partial gene sequences of uncultured and isolated *Alistipes* or *Coprococcus* from the humans' intestinal tracts. Their specificity was verified in silico using BLAST (http://blast.ncbi.nlm.nih.gov/) and ProbeMatch from the Ribosomal Database Project (RDP) (https://rdp.cme.msu.edu/). For experimental validation, DNAs extracted from *Bacteroides thetaiotaomicron* ATCC29148, Prevotella copri DSM18205, Lactobacillus rhamnosus GG, and Bifidobacterium bifidum MIMBB75 were used.

Supplementary Table 3. Liver histology by disease group

**Steatosis (% hepatocytes)** *Median (min, max)*	**HC (n=16)**	**SS (n=15)**	**NASH (n=24)**
	0 (0, 2.5)	15 (5, 70)	40 (10, 90)
**Fibrosis Stage** *Proportion of subjects (count)*	**HC (n=13)**	**SS (n=15)**	**NASH (n=24)**
0	77% (10)	67% (10)	12.5% (3)
1	23% (3)	20% (3)	25% (6)
2	0% (0)	13% (2)	33% (8)
3	0% (0)	0% (0)	12.5% (3)
4	0% (0)	0% (0)	17% (4)
**Lobular Inflammation Intensity** *Proportion of subjects (count)*	**HC (n=14)**	**SS (n=15)**	**NASH (n=24)**
0	100% (14)	87% (13)	4% (1)
1	0% (0)	13% (2)	67% (16)
2	0% (0)	0% (0)	21% (5)
3	0% (0)	0% (0)	8% (2)
**Total Inflammation**† *Proportion of subjects (count)*	**HC (n=10)**	**SS (n=5)**	**NASH (n=12)**
None	100% (10)	60% (3)	0% (0)
Mild	0% (0)	40% (2)	33% (4)
Moderate	0% (0)	0% (0)	67% (8)
Severe	0% (0)	0% (0)	0% (0)
**Ballooning Intensity** *Proportion of subjects (count)*	**HC (n=15)**	**SS (n=15)**	**NASH (n=23)**
None	100% (15)	100% (15)	4% (1)
Mild	0% (0)	0% (0)	61% (14)
Obvious	0% (0)	0% (0)	35% (8)
**NAFLD Activity Score** *Median (min, max)*	**HC (n=14)**	**SS (n=15)**	**NASH (n=24)**
0-8	0 (0, 0)	1 (1, 4)	4.5 (3, 8)

† This measure was not consistently recorded in all patients.
HC: healthy controls, SS: simple steatosis, NASH: nonalcoholic steatohepatitis

Supplementary Table 4. Reported physical activity by disease group

Variable	HC (n=22)	SS (n=11)	NASH (n=19)	p-value
Total Activity (average activity units† per day)	7.3 (4.9, 14.1)	6.2 (1.8, 12.0)	6.4 (3.7, 11.5)	0.7
Strenuous Activity (average minutes/day)	2.1 (0, 33.3)	0 (0, 4.3)	0 (0, 6.4)	0.15
Very Strenuous Activity (average minutes/day)	0 (0, 0)	0 (0, 0)	0 (0, 0)	0.19
Strenuous + Very Strenuous Activity (average minutes/day)	21.8 (0, 42.9)	0 (0, 4.3)	0 (0, 10)	0.053

Values are expressed as median (1st quartile, 3rd quartile) for non-normally distributed variables.
†1 activity unit = 30 minutes mild, 20 minutes moderate, 10 minutes strenuous, or 5 minutes very strenuous activity. This is a validated method for measuring physical activity level [5].
HC: healthy controls, SS: simple steatosis, NASH: nonalcoholic steatohepatitis

Supplementary Table 5. Environmental questionnaire results

**Variable**	**n**	**HC**	**n**	**SS**	**n**	**NASH**	**p-value**
Born in Canada	22	86% (19)[ab]	9	0% (0)[a]	16	31% (5)[b]	*<0.0001*
Breastfed	20	25% (5)	7	14% (1)	10	90% (9)	0.7
International Travel	20	30% (6)	8	37.5% (3)	10	10% (1)	0.4
Pets at Home	20	65% (13)	7	14% (1)	10	40% (4)	*0.047*

Values are expressed as proportion of patients (count).
Values with the same superscript are significantly different
HC: healthy controls, SS: simple steatosis, NASH: nonalcoholic steatohepatitis

Supplementary Table 6. Reported dietary intake: Energy and Macronutrients

Variable	HC (n=23)	SS (n=10)	NASH (n=20)	p-value
Energy Intake (kcal/kg/day)	25 (22, 33)[a]	24 (20, 30)	18 (13, 21)[a]	*0.001*
Energy Intake (kcal/day)	1834 (1679, 2418)	1844 (1455, 2176)	1626 (1176, 2055)	0.10
Fat-derived Energy (%)	35.5 (29.2, 40.0)	28.9 (26.6, 31.5)	32.8 (27.6, 38.9)	0.17
Protein-derived Energy (%)	16.9 (15.4, 20.0)	20.8 (18.2, 21.9)	18.9 (17.0, 21.4)	*0.041*[†]
Carbohydrate-derived Energy (%)	48.2 (43.9, 54.8)	51.0 (44.4, 56.3)	47.7 (39.2, 51.3)	0.5
Saturated Fat-derived Energy (%)	11.2 (9.6, 12.6)	7.9 (7.0, 11.4)	11.0 (9.8, 11.7)	0.17
Cholesterol (mg/day)	265 (170, 335)	239 (119, 354)	207 (146, 356)	
Cholesterol (mg/kcal)	0.13 (0.09, 0.15)	0.13 (0.10, 0.16)	0.14 (0.10, 0.21)	0.6
Fibre (g/day)	24.0 (18.4, 27.0)[a]	17.6 (13.6, 26.8)	17.3 (12.6, 20.6)[a]	
Fibre (g/kcal)	0.01 (0.01, 0.01)	0.01 (0.01, 0.02)	0.01 (0.01, 0.01)	0.5
Sugar-derived Energy (%)	19.4 (13.7, 24.5)	14.6 (10.1, 18.2)	14.7 (9.6, 20.0)	0.19
Omega 6:3	0.15 (0.12, 0.21)	0.18 (0.13, 0.27)	0.15 (0.12, 0.22)	0.8

Values are expressed as median (1st quartile, 3rd quartile).
Values with the same superscript are significantly different.
† Despite significant comparison with Krusal-Wallis test, pairwise comparisons did not reach significance.
HC: healthy controls, SS: simple steatosis, NASH: nonalcoholic steatohepatitis

Supplementary Table 7. Reported dietary intake: Micronutrients

Variable	HC (n=23)	SS (n=10)	NASH (n=20)	p-value
Vitamin A (IU/day)	8553 (4125, 15918)	6785 (5093, 12014)	8514 (6711, 13635)	
Vitamin A (IU/1000 kcal)	4.89 (2.55, 9.33)	4.94 (2.92, 7.41)	6.47 (2.42, 12.20)	0.5
Vitamin C (mg/day)	145 (90, 231)	108 (61, 132)	128 (69, 218)	
Vitamin C (mg/1000 kcal)	0.08 (0.05, 0.12)	0.06 (0.04, 0.08)	0.08 (0.04, 0.18)	0.6
Choline (mg/day)	228 (181, 284)	212 (165, 303)	189 (119, 258)	
Choline (mg/1000 kcal)	0.12 (0.09, 0.15)	0.12 (0.09, 0.16)	0.12 (0.10, 0.16)	0.9
Calcium (mg/day)	747 (638, 1269)	657 (437, 871)	509 (354, 1031)	
Calcium (mg/1000 kcal)	0.44 (0.30, 0.62)	0.33 (0.27, 0.60)	0.32 (0.21, 0.70)	0.4
Iron (mg/day)	14.5 (11.3, 19.8)	15.2 (13.3, 20.2)	10.7 (8.9, 15.7)	
Iron (mg/1000 kcal)	0.01 (0.01, 0.01)	0.01 (0.01, 0.01)	0.01 (0.01, 0.01)	0.17
Sodium (mg/day)	2460 (2144, 3400)	1971 (1620, 3290)	2199 (1529, 2709)	
Sodium (mg/1000 kcal)	1.37 (1.05, 1,82)	1.38 (0.92, 1.72)	1.36 (1.09, 1.66)	0.7

Values are expressed as median (1st quartile, 3rd quartile) for non-normally distributed variables.
HC: healthy controls, SS: simple steatosis, NASH: nonalcoholic steatohepatitis

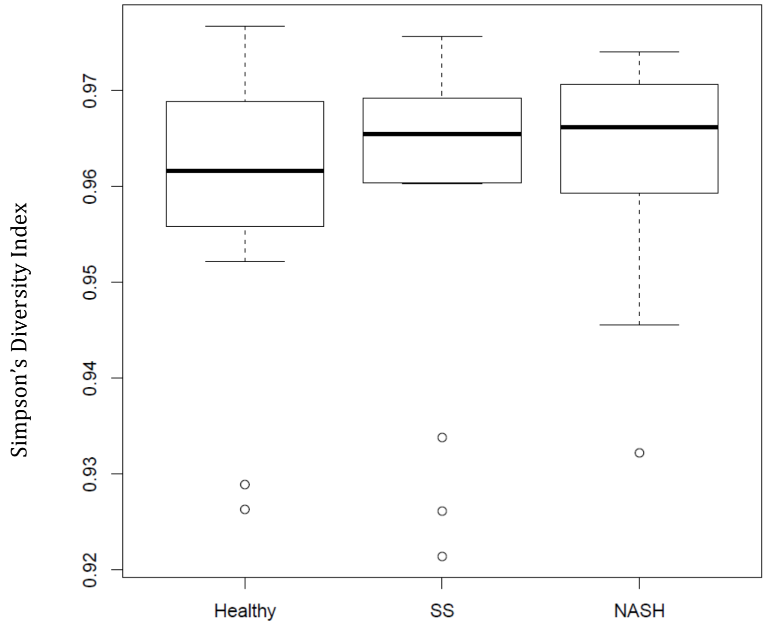

Supplementary Figure 1. Simpson's diversity index in healthy control, simple steatosis (SS), and nonalcoholic steatohepatitis (NASH) groups; OTU level data filtered at 1%

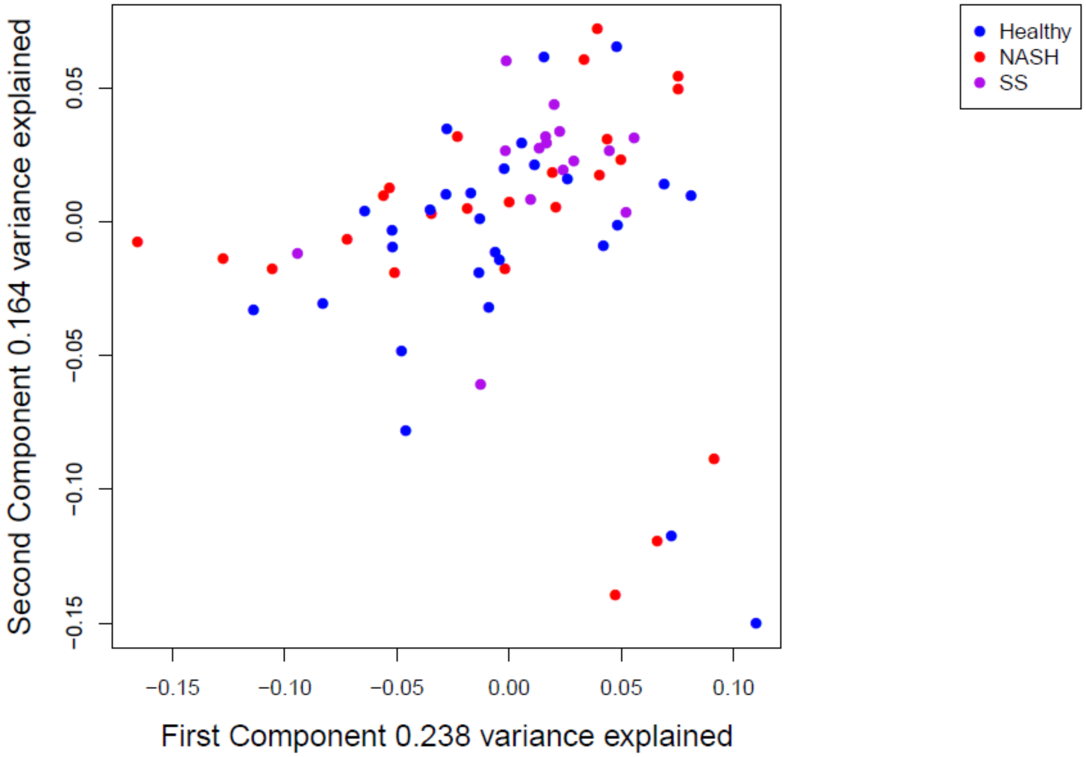

Supplementary Figure 2. Principal coordinate analysis, 1st and 2nd components, based on weighted UniFrac distance matrix for healthy control, simple steatosis (SS), and nonalcoholic steatohepatitis (NASH) groups.

Supplementary Table 8. Spearman correlation coefficients between liver histology parameters and bacterial groups quantified by qPCR. Restricted to NAFLD group (n=38)

correlation coefficient *p-value*	*% steatosis*	*lobular inflammatio n*	*balloonin g*	*zone 3 fibrosis*	*fibrosis*	*ballooning intensity*	*NAS*
Coprococcus	0.125<br>0.5	0.147<br>0.4	0.053<br>0.7	0.239<br>0.15	0.150<br>0.4	0.072<br>0.7	0.180<br>0.3
F. prausnitzii	0.154<br>0.4	0.186<br>0.3	-0.010<br>1.0	0.020<br>0.9	-0.079<br>0.6	-0.175<br>0.3	0.126<br>0.5
Ruminococcus	-0.106<br>0.5	-0.326<br>0.043	-0.157<br>0.3	-0.166<br>0.3	-0.092<br>0.6	-0.135<br>0.4	-0.219<br>0.18

NAS = NAFLD Activity Score

Supplementary Table 9. Spearman correlation coefficients between concentration of selected bacterial products and bacterial groups quantified by qPCR.

correlation coefficient<br>*p-value*	F.prausnitzii<br>Fecal n=65<br>Serum n=58	Ruminococcus<br>Fecal n=66<br>Serum n=59	Coprococcus<br>Fecal n=65<br>Serum n=58
Fecal Butyric Acid	0.013<br>0.9	-0.235<br>0.057	-0.033<br>0.8
Fecal Acetic Acid	-0.073<br>0.073	-0.312<br>0.011*	N/A
Fecal Propionate	N/A	-0.302<br>0.014*	-0.096<br>0.4
Fecal L Lactic Acid	N/A	-0.126<br>0.3	0.043<br>0.7
Fecal Formic Acid	N/A	0.027<br>0.8	0.058<br>0.6
Serum Acid Acid	0.109<br>0.4	0.014<br>0.9	N/A
Serum Isobutyric Acid	-0.117<br>0.4	-0.248<br>0.058	0.069<br>0.6
Serum 2-hydroxybutyrate	-0.285<br>0.030	-0.223<br>0.090	-0.007<br>1.0
Serum Formic Acid	N/A	-0.095<br>0.5	0.067<br>0.6
Serum L Lactic Acid	N/A	-0.317<br>0.015*	-0.151<br>0.3
Serum Ethanol	N/A	0.059<br>0.7	N/A

*The p-values were not controlled for multiple comparisons, so the correlations with p<0.05 are likely to be due to Type I error.

REFERENCES

1 Smyth, G. K. Linear models and empirical bayes methods for assessing differential expression in microarray experiments. *Statistical applications in genetics and molecular biology* **3**, Article3, doi:10.2202/1544-6115.1027 (2004).

2 Paulson, J. N., Stine, O. C., Bravo, H. C. & Pop, M. Differential abundance analysis for microbial marker-gene surveys. *Nature methods* **10**, 1200-1202, doi:10.1038/nmeth.2658 (2013).

3 White, J. R., Nagarajan, N. & Pop, M. Statistical methods for detecting differentially abundant features in clinical metagenomic samples. *PLoS Comput Biol* **5**, e1000352, doi:10.1371/journal.pcbi.1000352 (2009).

4 Benjamini, Y. & Hochberg, Y. Controlling the false discovery rate: a practical and powerful approach to multiple testing. *J R Stat Soc Ser B* **57**, 289-300 (1995).

5 Pan, X. R. *et al.* Effects of diet and exercise in preventing NIDDM in people with impaired glucose tolerance. The Da Qing IGT and Diabetes Study. *Diabetes Care* **20**, 537-544 (1997).
